# Alkaptonuria presenting as lumbar degenerative disease: A case report and literature review

**DOI:** 10.1097/MD.0000000000041283

**Published:** 2025-01-17

**Authors:** Peiming Sang, Yanyan Ma, Jun Yang, Fan He, Jingyan Chen, Xie Zhang, Binhui Chen, Ying Cai, Zhenjing Chen

**Affiliations:** a Ningbo Medical Center LiHuiLi Hospital, Ningbo, Zhejiang, PR China; b The Affiliated LiHuiLi Hospital of Ningbo University, Ningbo, Zhejiang, PR China; c Department of Cytopathology, Ningbo Clinical Pathology Diagnosis Center, Ningbo, PR China.

**Keywords:** alkaptonuria, back pain, black disk, black urine, ochronosis

## Abstract

**Rationale:**

Alkaptonuria (AKU) is a rare, inherited metabolic disease caused by deficient activity of homogentisic acid oxidase, leading to the accumulation of homogentisic acid and its oxidized product, benzoquinone acetic acid. These compounds cause black discoloration of cartilage, degeneration, inflammation, and calcification of intervertebral disks and large joints, resulting in pain and impaired quality of life. Despite its debilitating effects, there are no curative treatments for AKU, and management remains supportive. This study aims to contribute to the limited literature on AKU-related spinal manifestations by reporting a case of lumbar degenerative disease in an AKU patient and highlighting surgical intervention as an effective treatment approach.

**Patient concerns:**

A 49-year-old woman presented with chronic lower back pain and the posterior side of right lower limb radiating pain for 1 year, worsening over the past week. Magnetic resonance imaging revealed lumbar spondylolisthesis at the L4/5 level and migrated lumbar disk herniation at the L5/S1 level.

**Interventions:**

The patient underwent transforaminal lumbar interbody fusion surgery at L4/5 and L5/S1. Intraoperatively, the resected disk material was black, with darkened intervertebral disks and cartilage endplates, distinct from the typical white appearance of degenerative disks. Surgical intervention included disk and cartilage endplate removal, insertion of cages with harvested autografts, and implantation of bilateral pedicle screws and rods.

**Diagnosis:**

AKU with lumbar degenerative disease.

**Outcome:**

The patient experienced resolution of pain postoperatively.

**Lessons:**

This case underscores the importance of recognizing AKU as a potential cause of lumbar degenerative disease and highlights transforaminal lumbar interbody fusion surgery as a viable treatment option for pain relief and improved functionality in affected individuals.

## 1. Introduction

Lumbar degenerative disease is a common cause of lower back pain, affecting roughly 1% to 3% of the population annually and most commonly involving those between 30 and 50 years of age.^[1]^ However, the pathogenesis of lumbar degenerative disease is complicated. Lumbar degenerative disease is most commonly idiopathic but can occur secondary to trauma or physiological disk degeneration from the natural aging process.^[1]^ Risk factors include obesity, smoking, diabetes, connective tissue disorders, and genetic predisposition.^[2]^

Alkaptonuria (AKU) is a rare cause of lumbar degenerative disease and a rare monogenic autosomal-recessive disease caused by dysfunction of protein metabolism.^[3,4]^ The incidence of AKU is 0.001%.^[5]^ The 3 major features of AKU are dark urine or urine that turns dark on standing, ochronosis (bluish-black pigmentation in connective tissue), and arthritis of the spine and larger joints.^[6]^ However, lumbar degenerative diseases such as disk herniation are rare characteristics of AKU.^[5]^ There are only a few surgically treated cases of AKU presenting with lumbar degenerative disease, which have been described in the literature.^[5,7–11]^

We present a rare case of AKU presenting with lumbar degenerative disease in which the patient has been operated on with transforaminal lumbar interbody fusion (TLIF). During surgery, the disk material removed was black, which was different from the color of the conventional disk intraoperatively. Through research, analysis, and reexamination, this patient was diagnosed with AKU, presenting as a lumbar degenerative disease.

## 2. Case presentation

A 49-year-old woman presented with chronic lower back pain and the posterior side of right lower limb radiating pain for 1 year, worsening over the past week. Her right leg pain was more severe than the lower back pain, which caused her walking distance to be 200 miles. Ten years earlier, she had seen the doctor due to intermittent dark urine; however, the reason was never determined. Therefore, she ignored the dark urine, which did not cause other symptoms. There was no previous medical or surgical history. A spine examination revealed the pain was at the lower lumbar spine.

**Figure 1.**
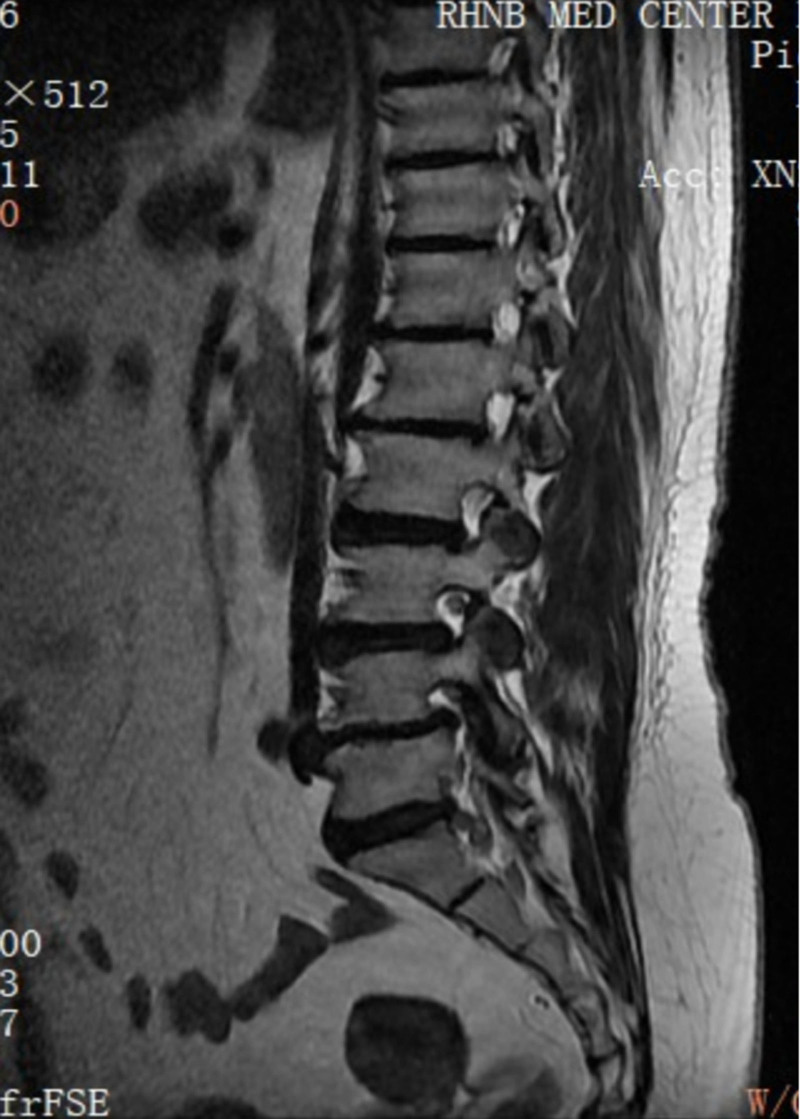
MRI sagittal view showing migrated lumbar disc herniation at the L5/S1 level. MRI = magnetic resonance imaging.

**Figure 2.**
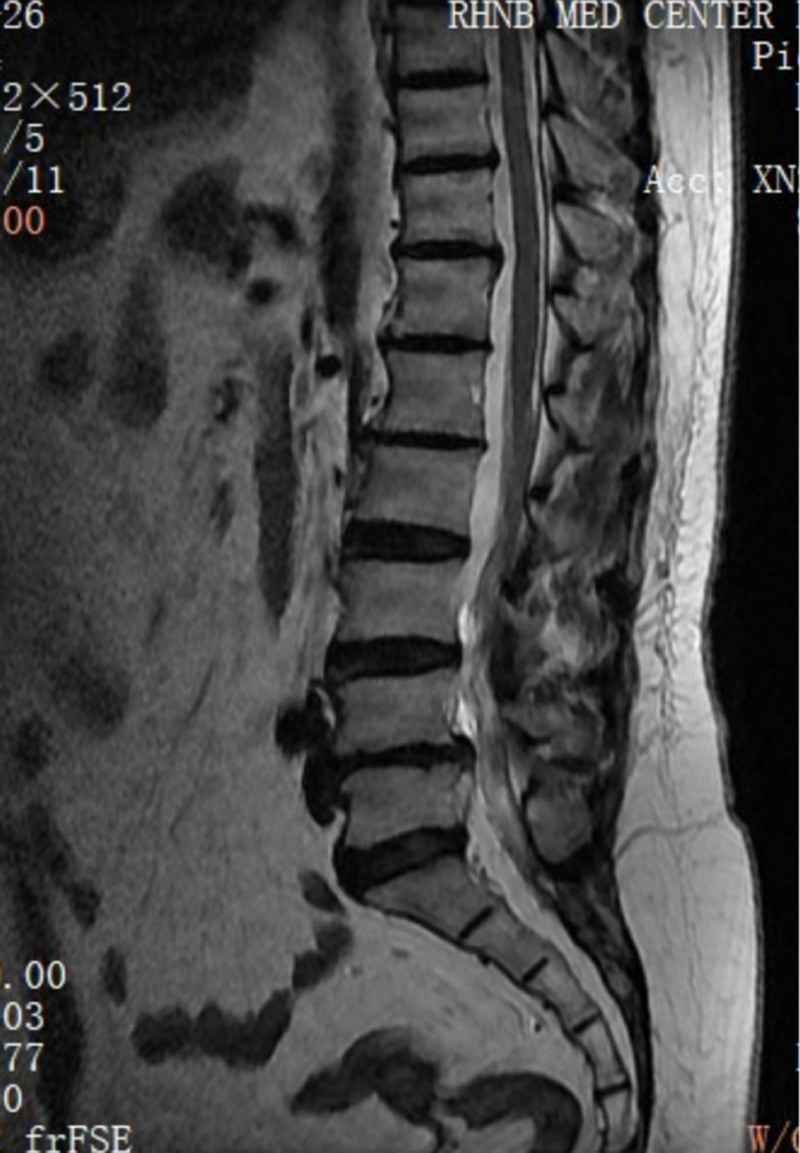
MRI sagittal view showing lumbar spondylolisthesis at the L4/5 level. MRI = magnetic resonance imaging.

**Figure 3.**
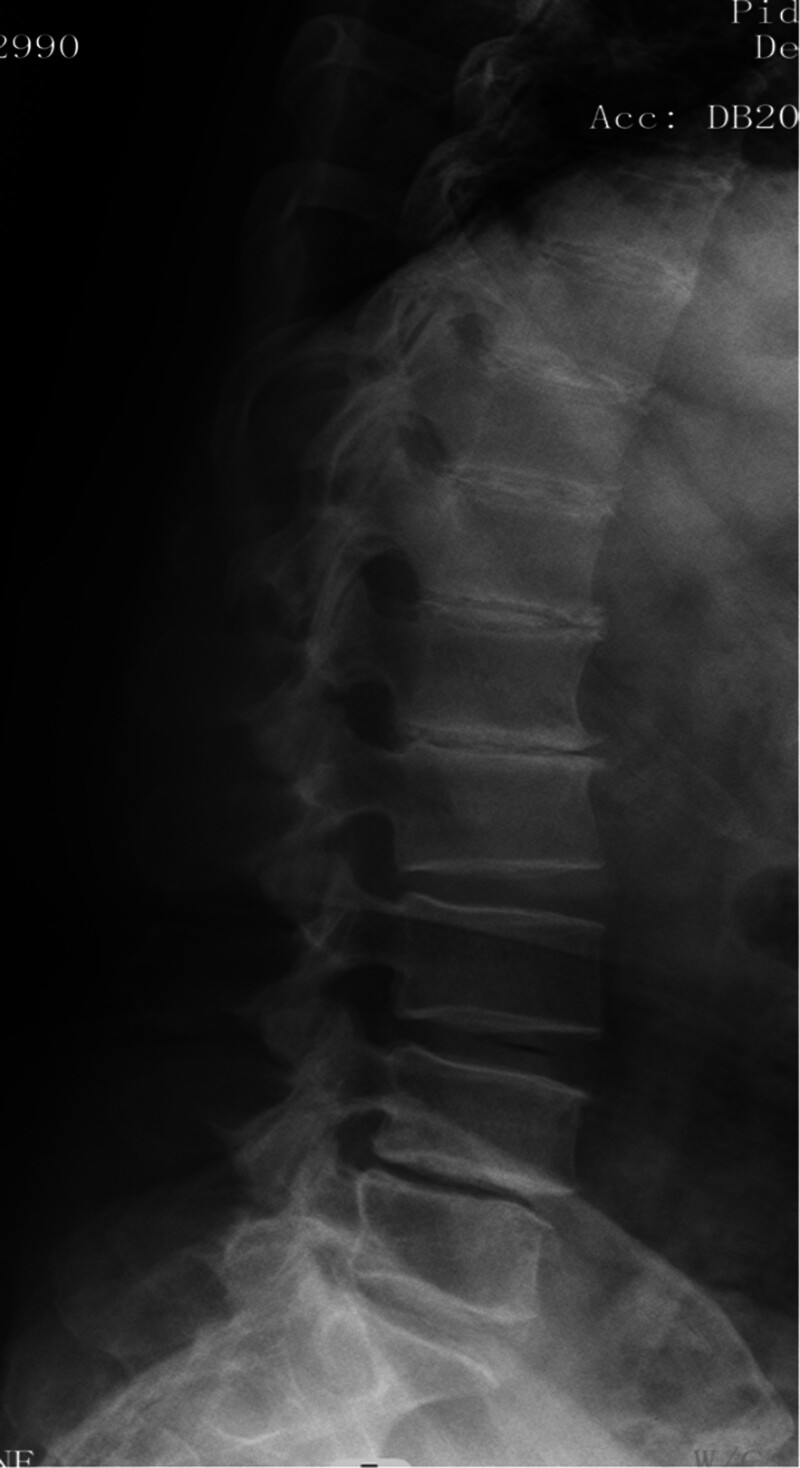
X-ray plain film showed intra-discal calcification or vacuum in multiple discs along with end plate sclerosis.

**Figure 4.**
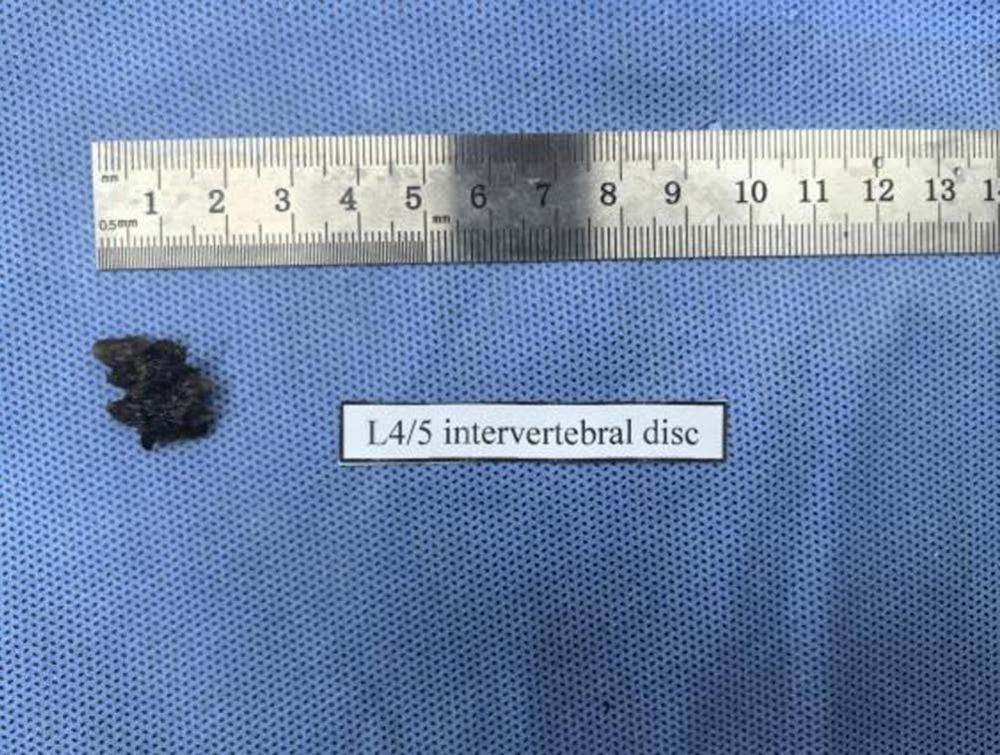
Intervertebral disc at the level of L4/5.

**Figure 5.**
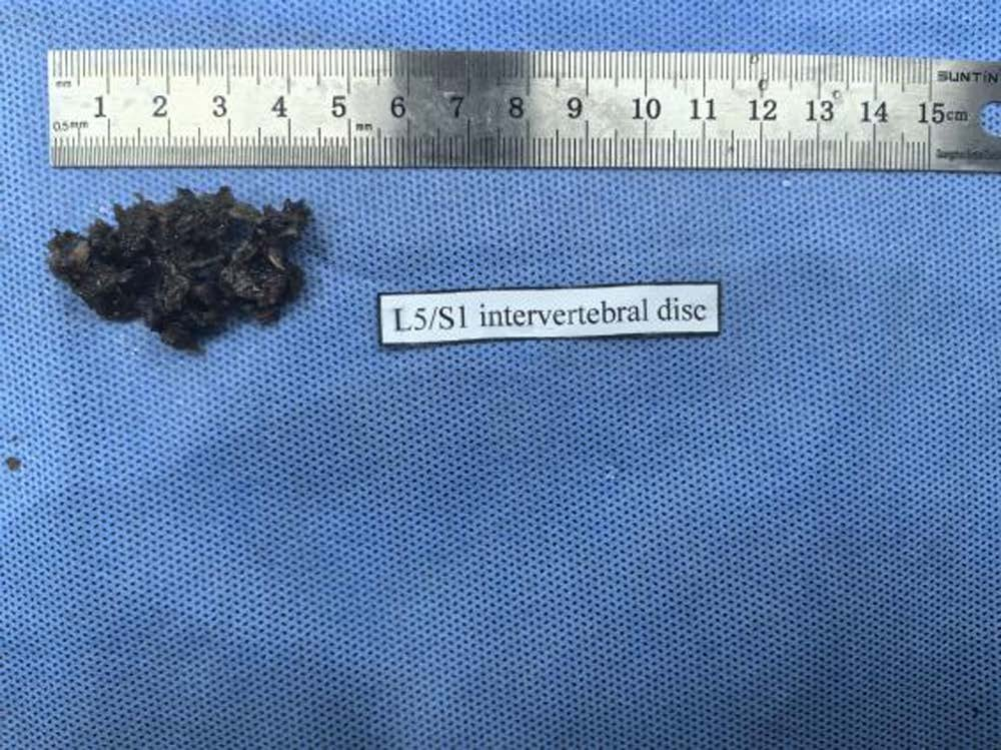
Intervertebral disc at the level of L5/S1.

**Figure 6.**
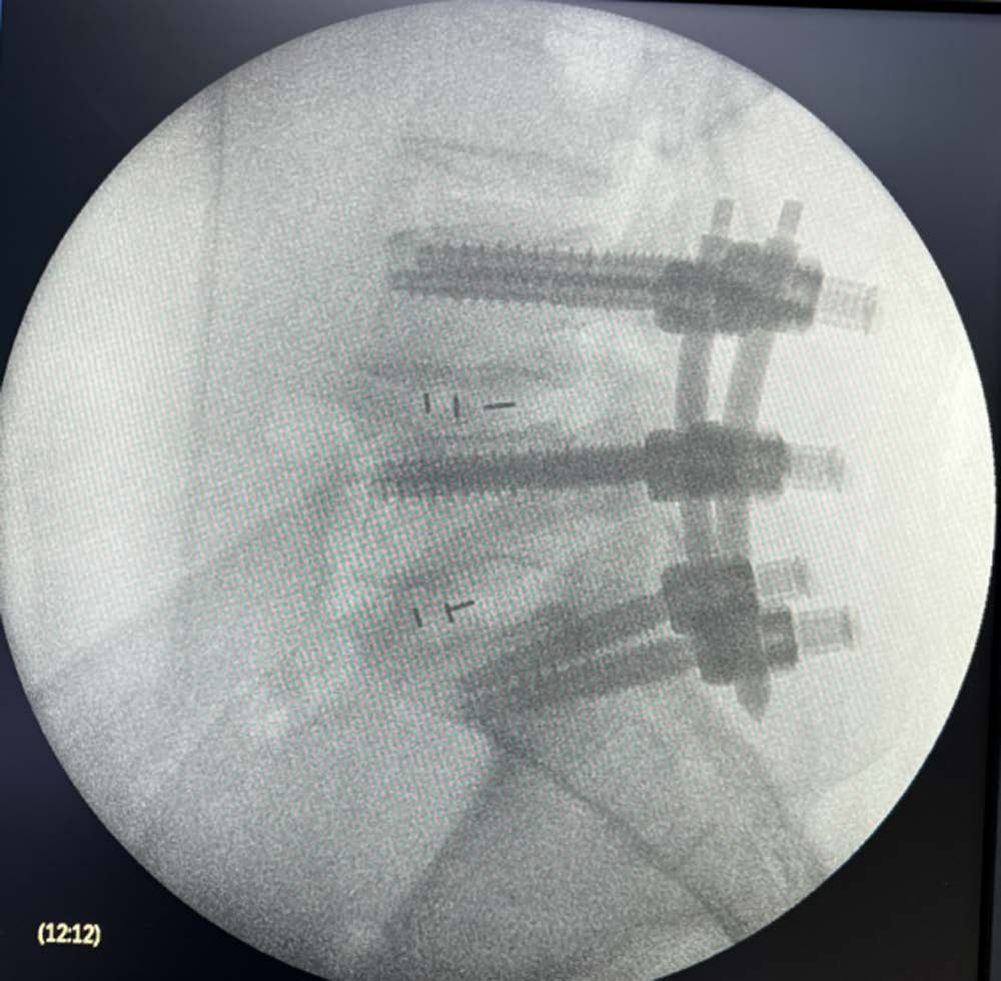
Spine radiograph with lateral view during the operation.

**Figure 7.**
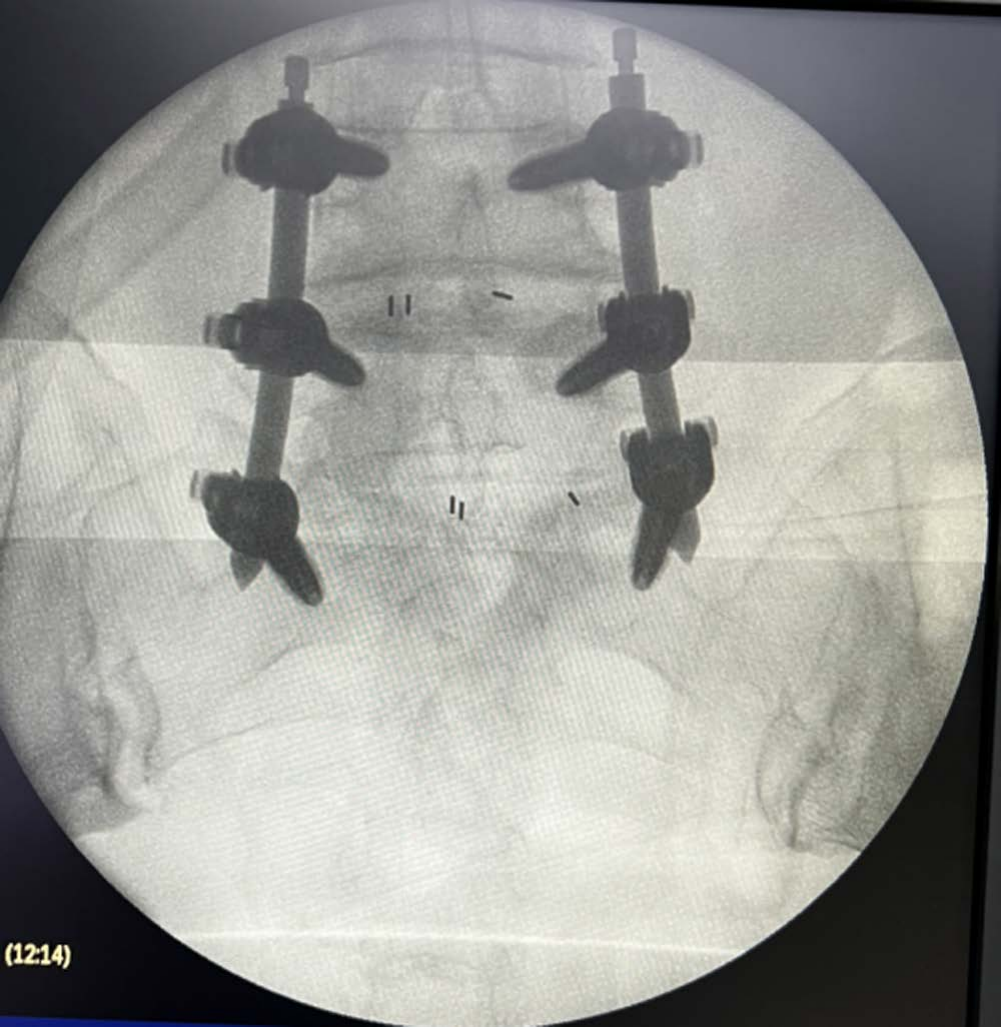
Spine radiograph with anteroposterior view during the operation.

Her neurological examination revealed that the myodynamia of lower limbs was normal and the straight leg raising test was negative bilaterally. The magnetic resonance imaging revealed lumbar spondylolisthesis at the L4/5 level and migrated lumbar disk herniation at the L5/S1 level (Figs. 1 and 2). A plain X-ray revealed intra-discal calcification or vacuum in multiple disks and end plate sclerosis (Fig. 3). There was lack of homogentisic acid testing in this case. After evaluating the Oswestry Disability Index and visual analog scale preoperatively, the patient was advised of an operation; she underwent a right-sided TLIF at L4/5 and L5/S1 levels. Intraoperative findings showed that migrated lumbar disk herniation of L5/S1 segment located at the space between the posterior edge of S1 vertebrae and the front of S1 nerve root, and there was lumbar spondylolisthesis at the L4/5 level. The color of resected disk material was black, which was different from conventional tissue (Figs. 4 and 5). The intervertebral space revealed dark tissue in the intervertebral disks and vertebral endplates, unlike degenerative disks with white color. The disks and cartilage endplates were obliterated. The cages with harvested autograft were inserted into the intervertebral space, and the bilateral pedicle screws and rods were implanted. A C-arm X-ray machine obtained intraoperative images (Figs. 6 and 7). After the procedure, the pain in her right leg disappeared immediately. Two weeks after surgery, pain associated with the wound located in the lower back disappeared. There were no intraoperative or postoperative complications. The patient would return to normal over 1 month.

**Figure 8.**
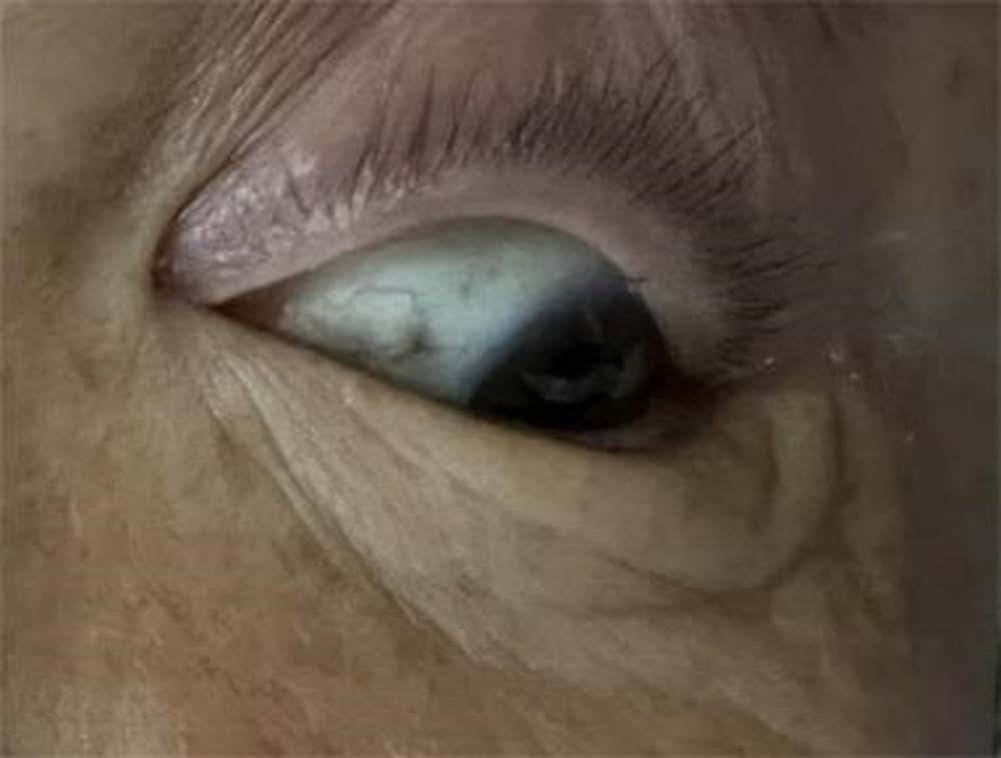
Brownish-black pigmentation of the sclera in left eye.

**Figure 9.**
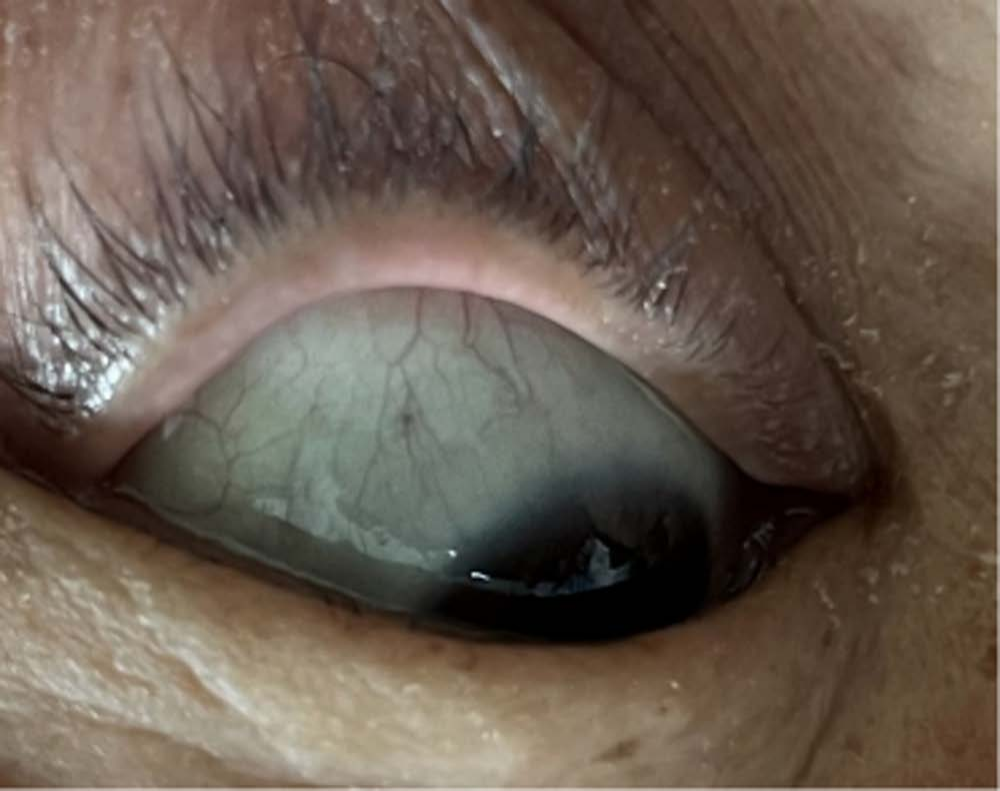
Brownish-black pigmentation of the sclera in right eye.

**Figure 10.**
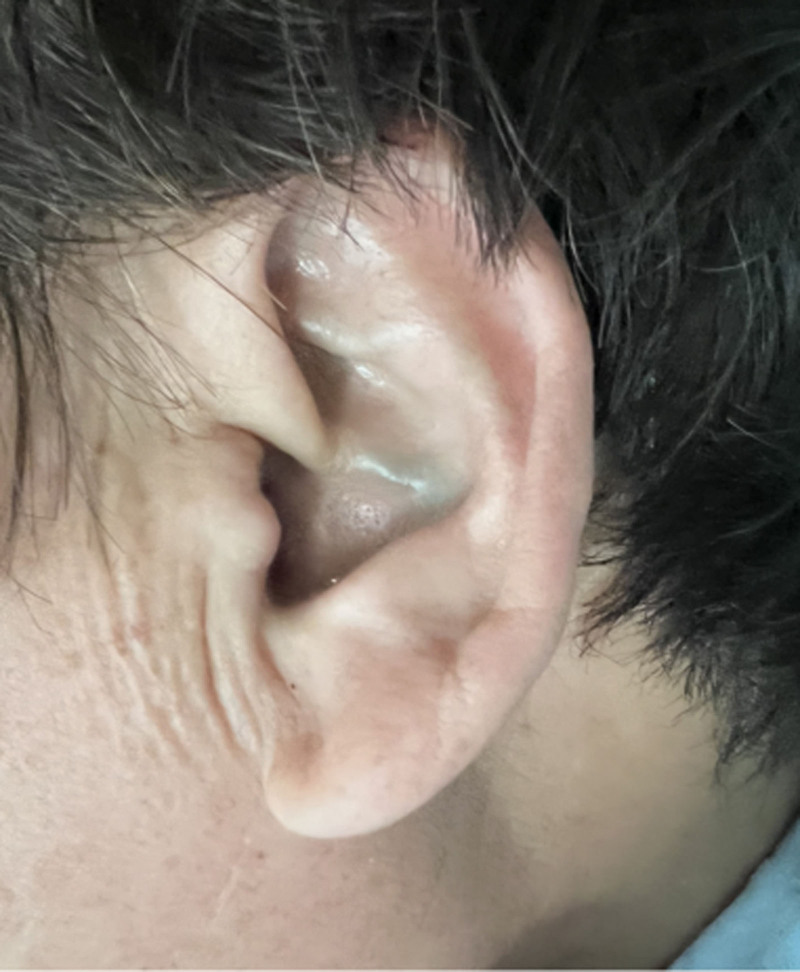
Grey pigmentation of cartilage in left ear.

**Figure 11.**
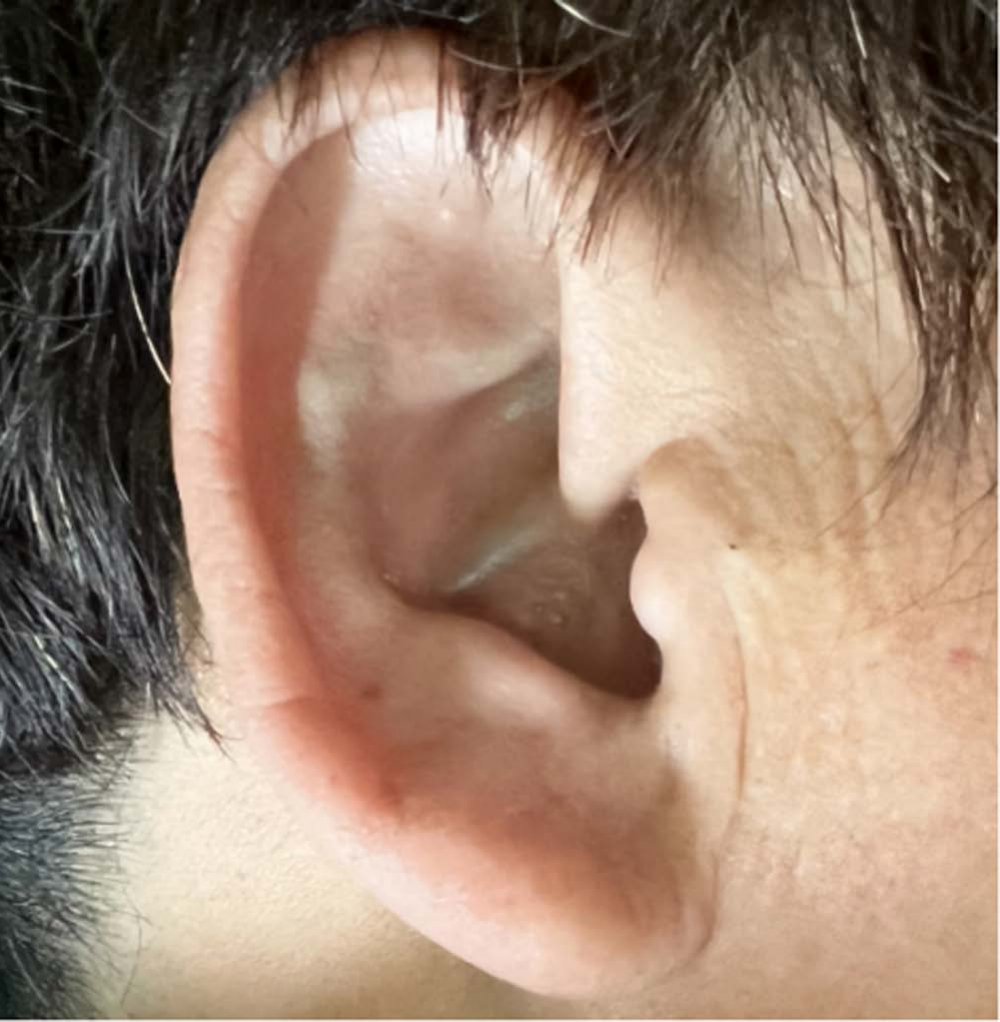
Grey pigmentation of cartilage in right ear.

**Figure 12.**
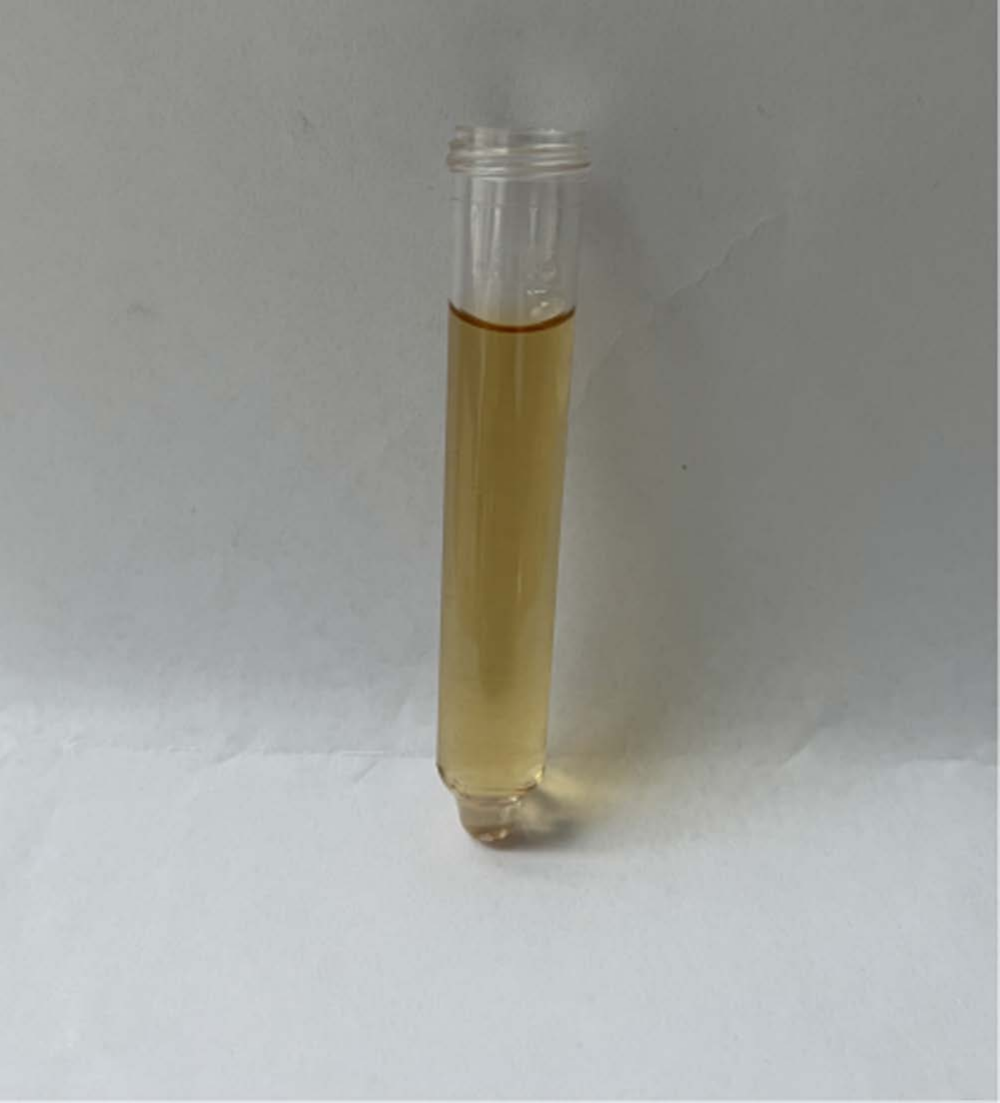
The color of the patient^’^ urine.

**Figure 13.**
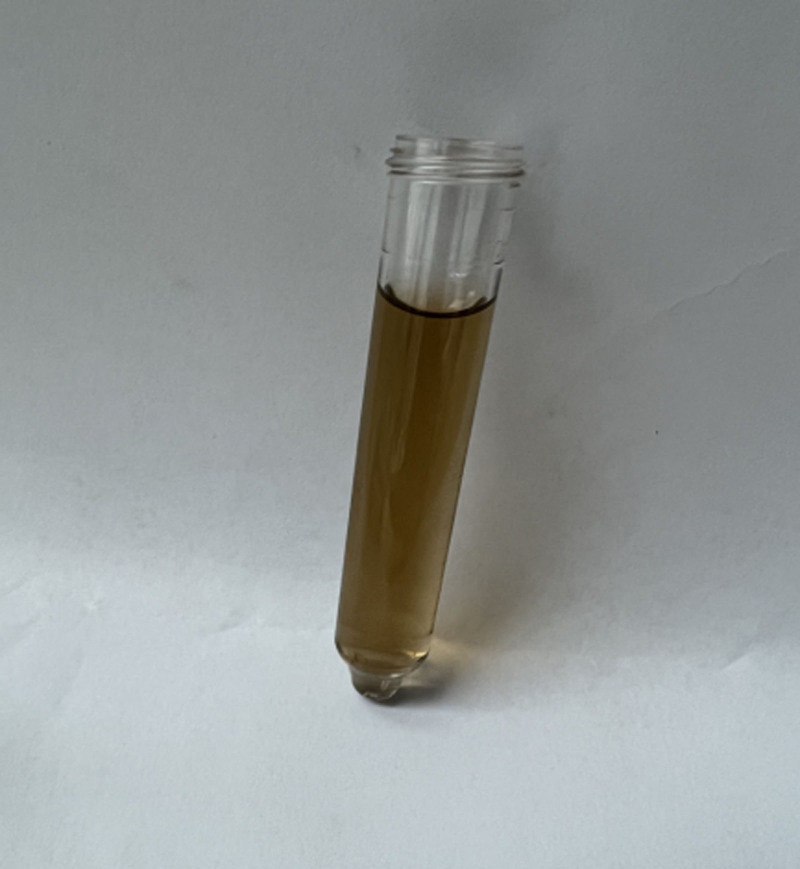
The patient’ urine turned dark on standing for 3 hours.

Through reexamination after surgery, brownish-black pigmentation of the sclera in her eyes (Figs. 8 and 9) and gray pigmentation of cartilage in the ears (Figs. 10 and 11) were observed. The color of the patient’s urine was also observed, and it turned dark after standing for 3 hours (Figs. 12 and 13). These characteristics led to the diagnosis of AKU in this case.

## 3. Discussion

AKU is a rare genetic inborn disease that affects protein metabolism; it is caused by a deficiency of homogentisate 1,2-dioxygenase, an enzyme that converts homogentisic acid to maleylacetoacetic acid in the tyrosine degradation pathway.^[6]^ AKU arises from homozygous or compound heterozygous mutations in the homogentisate 1,2-dioxygenase gene, and there have been 212 unique AKU mutations.^[12]^ AKU mutations are distributed throughout the homogentisate 1,2-dioxygenase gene with some prevalence in exons 3, 6, 7, 8, and 13.^[13]^

There is no consensus about clinical diagnostic criteria for AKU. However, the accumulation of homogentisic acid caused by a block in tyrosine metabolism can cause painful multisystemic diseases that reduce the quality of life. It has some features of AKU, such as dark urine or urine that turn dark on standing, ochronosis (bluish-back pigmentation of connective tissue), and arthritis.^[6]^ The severity and incidence rate of symptoms in AKU disease were associated with age. There were few signs and symptoms in the young,^[14]^ with symptoms appearing around age 30 years and progressively increasing in severity with age.^[15–17]^ Involvement of the spine usually appeared in the third decade.^[6]^ Intervertebral calcification at numerous levels and vacuum phenomena with radiolucent gas collections were typical radiologic findings indicating severe degeneration.^[18]^ In one large series, low back pain was observed before the age of 30 years in 49% of individuals and before the age of 40 years in 94%.^[19]^

In our case, some special clinical features played an essential role in diagnosing AKU. The lumbar spinal disks were black, confirmed by surgery. There was black pigmentation of the sclera and ear pinna. The urine color would become black after exposure to air for 3 hours. Plain films of the lumbar spine showed intra-discal calcification and radiolucent gas collections in multiple segments of disks, along with end plate sclerosis, which showed severe degeneration of the lumbar spine. The above mentioned factors were the accumulation of homogentisic acid and the expression of AKU.

There are no effective treatments for AKU. Due to the diversity of homogentisate 1,2-dioxygenase mutations in AKU, a personalized approach to gene therapy is probably not feasible.^[20]^ Some studies^[3,6,12]^ showed that the method of treating AKU depends on the patients, symptoms and genes; however, enzyme replacement therapies remain in the future.

The treatment of AKU is supportive and palliative. The therapy included lifestyle modifications, pharmacological management, and surgical intervention. Knee, hip, and shoulder replacement surgeries were options for managing significant arthritis.^[6]^ In a review of case reports requiring surgical intervention, the most frequent to least frequent causes were compression of cervical, thoracic, and lumbar dural sac or nerve root.^[21]^ The treatment for spinal degenerative disease in AKU is multi-faceted, focusing on the decompression of the dural sac or nerve root, stability of the spine, and maintaining spinal alignment. If the patients present only with compression and suffer from leg pain, decompression surgery such as discectomy is advised. However, if the patients with lower back and leg pain have undergone the compression of the dural sac or nerve root, the instability of the spine, the surgery of TLIF is suggested to decompress the dural sac or nerve root, preventing further collapse and stabilizing the spine.

In this case, the patient complained of chronic lower back pain and right lower limb radiating pain for 1 year, and the pain aggravated for 1 week. The magnetic resonance imaging revealed lumbar spondylolisthesis at the L4/5 level and migrated lumbar disk herniation at the L5/S1 level. The surgery was appropriate.

This study has several limitations that must be acknowledged. First, as a single case report, the results cannot be generalized to the broader population of AKU patients with spinal manifestations. Additionally, the retrospective analysis of clinical features and surgical outcomes limits the ability to draw causal inferences. Imaging findings and surgical observations were pivotal in diagnosis, but molecular or genetic testing was not performed to confirm the specific mutation. Lastly, the long-term efficacy and durability of the surgical intervention remain uncertain, requiring further follow-up and additional studies involving larger cohorts. Future research should address these gaps by incorporating genetic analysis, exploring nonsurgical management strategies, and evaluating outcomes through multicenter longitudinal studies.

## 4. Conclusions

This study underscores the importance of clinical vigilance in diagnosing AKU, particularly through urine analysis and intraoperative observations during spinal surgeries. While surgical interventions offer symptomatic relief, the findings stress the need for a multidisciplinary approach, including further research into potential disease-modifying treatments.

## Author contributions

**Formal analysis:** Peiming Sang, Yanyan Ma, Ying Cai, Zhenjing Chen.

**Methodology:** Jingyan Chen.

**Resources:** Jun Yang, Fan He.

**Supervision:** Fan He, Jingyan Chen, Binhui Chen.

**Writing – original draft:** Peiming Sang, Yanyan Ma.

**Writing – review & editing:** Peiming Sang, Yanyan Ma, Xie Zhang.
